# Pharmacokinetics in the Fontan circulation: a multi-organ puzzle

**DOI:** 10.1093/ehjcvp/pvag058

**Published:** 2026-08-10

**Authors:** Panayotis K Vlachakis, Maria Drakopoulou, Panagiotis Theofilis, Paschalis Karakasis, Ioannis Leontsinis, Konstantinos Tsioufis, Konstantinos Toutouzas

**Affiliations:** First Department of Cardiology, Hippokration General Hospital of Athens, National and Kapodistrian University of Athens, 114 Vasilissis Sophias, Athens 11527, Greece; Unit of Structural Heart Diseases, First Department of Cardiology, Medical School, Hippocration General Hospital of Athens, National and Kapodistrian University of Athens, 114 Vasilissis Sophias, Athens 11527, Greece; First Department of Cardiology, Hippokration General Hospital of Athens, National and Kapodistrian University of Athens, 114 Vasilissis Sophias, Athens 11527, Greece; Unit of Structural Heart Diseases, First Department of Cardiology, Medical School, Hippocration General Hospital of Athens, National and Kapodistrian University of Athens, 114 Vasilissis Sophias, Athens 11527, Greece; First Department of Cardiology, Hippokration General Hospital of Athens, National and Kapodistrian University of Athens, 114 Vasilissis Sophias, Athens 11527, Greece; Unit of Structural Heart Diseases, First Department of Cardiology, Medical School, Hippocration General Hospital of Athens, National and Kapodistrian University of Athens, 114 Vasilissis Sophias, Athens 11527, Greece; Second Department of Cardiology, Hippokration General Hospital, Aristotle University of Thessaloniki, Thessaloniki 54642, Greece; First Department of Cardiology, Hippokration General Hospital of Athens, National and Kapodistrian University of Athens, 114 Vasilissis Sophias, Athens 11527, Greece; First Department of Cardiology, Hippokration General Hospital of Athens, National and Kapodistrian University of Athens, 114 Vasilissis Sophias, Athens 11527, Greece; Unit of Structural Heart Diseases, First Department of Cardiology, Medical School, Hippocration General Hospital of Athens, National and Kapodistrian University of Athens, 114 Vasilissis Sophias, Athens 11527, Greece; First Department of Cardiology, Hippokration General Hospital of Athens, National and Kapodistrian University of Athens, 114 Vasilissis Sophias, Athens 11527, Greece; Unit of Structural Heart Diseases, First Department of Cardiology, Medical School, Hippocration General Hospital of Athens, National and Kapodistrian University of Athens, 114 Vasilissis Sophias, Athens 11527, Greece

**Keywords:** Fontan circulation, Treatment, Pharmacokinetics, Congenital heart disease

## Abstract

Patients with Fontan circulation represent a pharmacologically distinct population in whom the haemodynamic consequences of single-ventricle physiology alter every phase of drug disposition in ways that current prescribing practice systematically fails to account for. Fontan-associated liver disease suppresses hepatic CYP enzyme activity and reduces first-pass metabolism, increasing bioavailability and systemic exposure of drugs. Hypoalbuminaemia from protein-losing enteropathy elevates the free fraction of highly protein-bound agents, rendering standard total drug concentration targets unreliable. Reduced cardiac output contracts the volume of distribution of hydrophilic drugs and diminishes renal perfusion, while creatinine-based glomerular filtration rate equations systematically overestimate true renal function due to reduced skeletal muscle mass, leading to inadequate dose reduction for renally cleared agents. These disturbances are predicted to affect the pharmacokinetics of every major cardiovascular drug class prescribed in this population, including anticoagulants, antiarrhythmics, neurohormonal antagonists, and pulmonary vasodilators, although direct Fontan-specific pharmacokinetic data remain scarce and confirm these effects for only a minority of agents, with dosing largely extrapolated from non-congenital populations. This review examines the mechanistic basis of pharmacokinetic alterations across drug disposition, evaluate available drug-class evidence, and outlines a research agenda including physiologically based pharmacokinetic modelling, registry-embedded studies, and dedicated inclusion of Fontan patients in future trials.

## Introduction

Congenital heart disease affects approximately 8 per 1000 live births, and surgical advances over the past five decades have converted what was once a disease of childhood mortality into a chronic adult condition.^1^ Among the most complex lesions, those characterized by a functional single ventricle culminate in the Fontan operation, which routes systemic venous return directly to the pulmonary circulation without a sub-pulmonary pump.^2^ More than 70 000 individuals worldwide now live with this physiology, the majority adults, a population growing at approximately 5% annually as surgical survival rates approach 90% in experienced centres.^3,4^

Heart failure (HF) has emerged as the dominant cause of late mortality in Fontan population surpassing sudden cardiac death in most modern registry analyses.^5,6^ Current guidelines acknowledge these therapeutic challenges but provide no specific drug recommendations, as evidence remains limited and practice relies heavily on extrapolation from non-Fontan cohorts.^7–9^ It translates directly into prescribing ‘uncertainty’, wide inter-centre variation in dug choices, and the distinct possibility that doses calibrated by biventricular acquired HF systematically over- and under-expose Fontan patients to medications whose narrow therapeutic index demands precision.

The pharmacokinetic (PK) basis of this problem is mechanistically coherent. The Fontan circulation generates a constellation of organ-level perturbations, including hepatic congestion, gut wall oedema, lymphatic hypertension leading to protein-losing enteropathy (PLE), and progressive cardiorenal dysfunction, that collectively alter absorption, distribution, metabolism, and excretion in a manner that parallels but does not duplicate either cirrhosis or acquired HF.^10^ These perturbations operate simultaneously, compound each other, and evolve over decades as the patient ages and organ injury accumulate. No existing pharmacological trial population reproduces this combination.

The aim of this review is to examine how the multi-organ consequences of Fontan palliation systematically alter drug pharmacokinetics across the four phases of disposition, to critically appraise the drug-class-specific evidence where it exists, and to identify where dosing practice, anchored in populations whose physiology differs fundamentally from this circulation, remains unsupported by Fontan-specific data

## The Fontan circulation: organ-system determinants of altered pharmacokinetics

**Figure 1:**
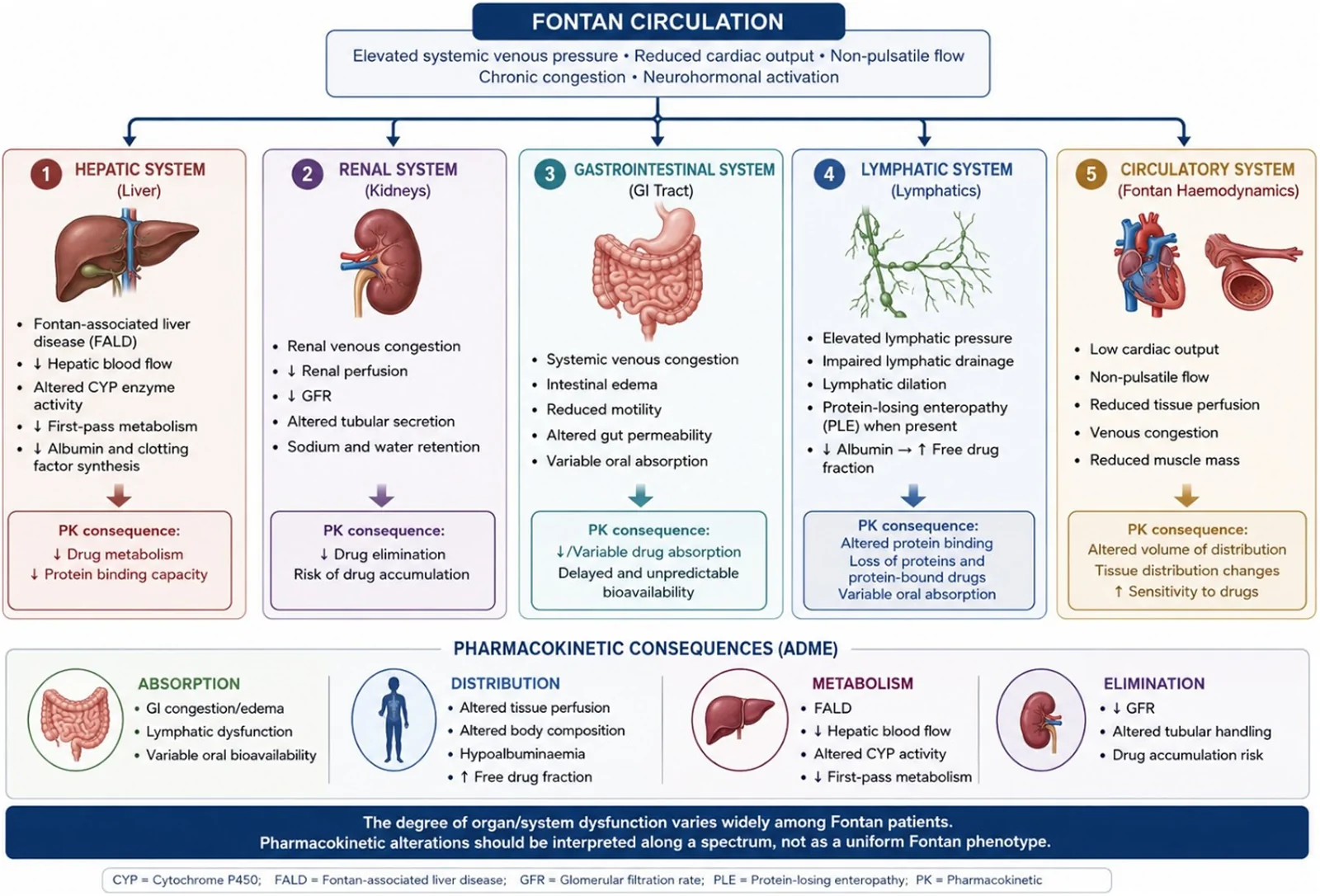
Pharmacokinetic consequences of the Fontan circulation. Created with BioRender.com.

To understand ‘why’ drug handling is abnormal in Fontan patients, it is necessary to appreciate the haemodynamic and organ-level consequences of passive pulmonary blood flow sustained over lifetime. Drug handling in Fontan patients is altered through five simultaneously operating organ-system and haemodynamic mechanisms involving the hepatic, renal, gastrointestinal, lymphatic, and circulatory systems. The severity of these abnormalities is highly heterogeneous across the Fontan population (*Figure 1*). Consequently, the pharmacokinetic perturbations discussed throughout this review should be interpreted as existing along a continuum that reflects the degree of individual organ-system dysfunction rather than representing a uniform Fontan phenotype.

Central venous pressure in the Fontan circuit is chronically elevated (typically 12–20 mmHg) because it must serve as the driving force for pulmonary perfusion in the absence of a right ventricular pump.^10^ This persistent venous hypertension transmits backwards through the hepatic, splanchnic, and renal venous systems, producing chronic congestion and progressive organ injury distinct from that seen in biventricular HF.^10,11^ Cardiac output (CO) is typically reduced to 60%–70% of predicted and fails to augment with demand, resulting in chronic under-perfusion of hepatic and renal parenchyma even in the absence of overt decompensation.^12^

Fontan-associated liver disease (FALD) is the most PK consequential downstream complication.^13^ Virtually all patients develop some degree of hepatic fibrosis within the first decade after surgery, with advanced fibrosis or cirrhosis documented in up to 43% at 30-years post-palliation.^14^ Histologically, FALD is characterized by sinusoidal dilation, centrilobular congestion, hepatocyte loss, and progressive perisinusoidal fibrosis.^15^ These features reduce functional hepatocyte mass and suppress cytochrome P450 enzyme expression, as confirmed by recent multiomic analyses, although direct PK quantification of CYP enzyme activity in Fontan patients remains lacking.^16,17^ The critical problem for clinicians is that standard liver function tests, including alanine aminotransferase and aspartate aminotransferase, frequently remain within normal limits in FALD despite histologically advanced disease, and transient elastography over-reads liver stiffness because venous congestion itself increases it independently of fibrosis severity.^14,18–20^ Normal aminotransferases therefore do not exclude impaired hepatic metabolic capacity in Fontan patient.

PLE complicates Fontan physiology in 4% to 13% of patients and represents a further PK hazard through its effect on plasma protein concentrations.^21^ The condition arises from chronic elevation of intestinal lymphatic pressure, which drives protein-rich lymph into the gut lumen, producing hypoalbuminemia, hypogammaglobulinaemia, and lymphopenia.^21^ Serum albumin may fall below 25 g/L, substantially increasing the unbound fraction of highly protein-bound drugs including warfarin, carvedilol and sacubitril/valsartan.^22–25^ The clinical consequence that demands emphasis is the systematic misinterpretation of total drug concentrations in this context: measured plasma levels appear low because total drug is low, but free drug concentration, which drives pharmacological effect and toxicity, may be normal or elevated.^26^ Dose escalation based on total level measurements in hypolbuminaemic patients risks toxicity while appearing numerically justified.^26^

Fontan-associated kidney disease, the third major PK modifier in Fontan physiology, is more prevalent than routine creatinine measurement suggests.^27,28^ Fontan patients characteristically have reduced skeletal muscle mass, partly from chronic low CO and partly from the protein-caloric deficit associated with PLA, which reduces skeletal muscle mass, lowers serum creatinine independently of renal function, and produces falsely reassuring estimated glomerular filtration rate (eGFR) values when creatinine-based equations are applied.^29,30^ Published comparisons of creatinine-based and cystatin-C-based eGFR in Fontan cohorts consistently show that the latter reveals impaired renal function in a substantial larger proportion of patients.^27,31^ Since many cardiovascular drugs rely on renal elimination systematic overestimation of GFR by creatinine-based equations translates directly into systematic overdosing of renal dose-reduction thresholds and risk of drug accumulation and toxicity.^32–34^ Notably, loop diuretics present the inverse problem: in impaired renal function, reduced tubular secretion of these highly protein-bound agents diminishes their delivery to the site of action in the thick ascending limb, producing diuretic resistance rather than drug accumulation.^35^

The five key organ-level and haemodynamic consequences that affect PK in patients with Fontan circulation are depicted in *Figure 1*. Accordingly, drug disposition in Fontan patients is altered across the four major PK pathways—absorption, distribution, metabolism, and elimination—each of which is influenced by the unique Fontan haemodynamics.

## Integrated pharmacokinetic consequences

### Absorption and distribution

The haemodynamic and anatomical features of the Fontan circulation create conditions that would be expected to alter both the absorption and distribution of orally administered cardiovascular drugs. Elevated systemic venous pressure is transmitted directly to the splanchnic bed, providing a plausible substrate for gut wall oedema and delayed or reduced oral drug absorption analogous to that documented in decompansted biventricular HF.^36–38^ For high first-pass drugs such as carvedilol, the hepatic congestion characteristic of FALD would be predicted to paradoxically increase systemic bioavailability by analogy with patients with hepatic cirrhosis, though this effect has not been directly measured in any Fontan cohort.^22,26,39,40^ Reduced CO would be expected to contract the apparent volume of distribution by limiting drug delivery to peripheral tissues, an assumption formally incorporated into the Fontan-specific phyiologically based pharmacokinetic (PBPK) model used to guide rivaroxaban dosing in the UNIVERSE programme.^41^ These predictions, while mechanistically coherent and consistent with evidence from analogous disease states, remain hypotheses: no Fontan-specific PK study has directly measured oral bioavailability or volume of distribution for any cardiovascular drug in this population, and they should not be treated as established pharmacokinetic findings. This represents a fundamental evidence gap: the dosing of most long-term cardiovascular medications in Fontan patients remains anchored in data from healthy subjects or biventricular HF cohorts whose physiological substrate differs substantially from the chronic, non-compensating Fontan circulation.

### Metabolism and elimination

#### Liver

The liver is the central PK organ, responsible for biotransformation of most cardiovascular drugs through cytochrome P450-mediated phase I reactions and glucuronyl transferase-mediated phase II conjugation.^42^ In Fontan patients, hepatic drug metabolism is compromised through two distinct and additive mechanisms: reduced hepatic blood flow, limiting drug delivery to metabolizing hepatocytes and preferentially affecting drugs with high hepatic extraction ratios, and reduced enzymatic capacity, affecting drugs whose metabolism depends on CYP enzyme content rather than blood flow.^14,16^ The Wilkinson-Shand hepatic extraction model provides the theoretical framework for understanding this distinction: drugs with extraction ratios above 70%, the so-called flow-limited drugs, have their clearance governed by hepatic perfusion and would be expected to accumulate when CO falls, while drugs with low extraction ratios are governed by intrinsic enzyme activity and would be expected to accumulate specifically when CYP abundance falls with FALD progression.^43,44^

Among the flow-limited drugs commonly used in Fontan patients, carvedilol deserves particular attention. Its oral bioavailability in healthy subjects is already low, approximately 25%, owing to extensive first-pass CYP2D6 and CYP2C9 metabolism, but rises substantially as hepatic blood flow falls and first-pass extraction is reduced.^39,40^ This mechanism predicts that a standard weight-based oral dose of carvedilol could produce two- to three-fold or greater peak plasma concentrations in a Fontan patient with reduced CO compared with an adult with normal biventricular function, a hypothesis consistent with, though not directly confirmed by, the excess hypotension and drug discontinuation rates observed in case series of neurohormonal blockade in complex congenital heart disease.^24,45,46^ A pharmacogenomic dimension compounds this: CYP2D6 polymorphisms, present in 7%–10% of European populations as poor metabolizers, further reduce carvedilol clearance and have not been characterized as a variable in any Fontan pharmacotherapy study.^47^

Among the capacity-limited drugs, amiodarone occupies a uniquely problematic position in Fontan pharmacology. It is simultaneously a substrate of CYP3A4 and an inhibitor of CYP3A4, CYP2C9, and CYP2D6, meaning its own FALD-driven accumulation would be expected to further suppress clearance of co-administered drugs including warfarin, bosentan, and several antiarrhythmics.^48–50^ Clinical series document side-effect rates exceeding 50%, predominantly thyroid dysfunction and hepatotoxicity, and amiodarone's hepatotoxic potential is expected to be magnified by pre-existing FALD.^51,52^ Despite this, amiodarone remains widely prescribed in Fontan patients with refractory atrial arrhythmias because the alternatives are mechanistically less appealing or similarly unstudied.^52^

#### Kidney

Renal elimination of cardiovascular drugs is systematically overestimated in Fontan patients because the creatinine-based equations embedded in clinical practice were derived from populations with normal muscle mass and conventional haemodynamics.^11,53^ Fontan patients exhibit chronic reduction in skeletal muscle mass from the combined effects of low CO, enteric protein loss, and the metabolic consequences of childhood single-ventricle physiology.^30^ The resulting reduction in creatinine production makes serum creatinine an unreliable surrogate for GFR, and the commonly used CKD-EPI and MDRD equations accordingly overestimate measured GFR by 15% to 20% in published comparisons.^31^ Cystatin C, a low-molecular-weight protein produced at a constant rate by all nucleated cells and filtered freely at the glomerulus, is significantly less influenced by muscle mass and provides a more accurate GFR estimate in Fontan patients.^28,54^ The clinical consequences of this systematic overestimation are most acute for renally cleared drugs with narrow therapeutic indices.

Organic anion transporter (OAT) 1 and OAT3, which mediate the tubular secretion of many drugs including furosemide, are substrate-saturable and can be competitively inhibited by endogenous organic anions that accumulate as GFR declines.^55^ In the context of Fontan physiology, where multiple organic acid substrates from impaired hepatic metabolism may compete for tubular secretion, the effective renal clearance of drugs dependent on OAT-mediated secretion may be substantially lower than predicted from GFR alone, though this mechanism has not been specifically investigated.^56^

## Drug-class-specific evidence

### Antithrombotic therapy

Thromboembolism is a major complication of the Fontan circulation, driven by chronically low-velocity non-pulsatile flow, endothelial dysfunction, prosthetic material, atrial arrhythmias, and coagulation abnormalities.^2^ In untreated cohorts, thromboembolic incidence reaches 3.5 per 100 patient-years, and large registry data, with a median follow-up of 14.4 years, show that an absence of antithrombotic therapy is associated with markedly increased mortality, particularly in hypoplastic left heart syndrome.^57,58^ These data shift the question from whether to treat to which agent to use, and whether Fontan-specific PK alterations are adequately considered in that choice.

Antiplatelet therapy with aspirin consists the most commonly used strategy. In a network meta-analysis of 21 studies and 26 546 patient-years, aspirin reduced thromboembolic events compared with no prophylaxis (incidence rate ratio 0.24; 95% CI 0.15–0.39) and demonstrated a favourable efficacy and safety profile, with low bleeding risk.^57^ From a PK standpoint, aspirin cannot be treated as haemodynamically inert in the hypoalbuninaemic Fontan patient. Its primary metabolite, salicylate, is approximately 90% albumin-bound, and in the setting of PLE-associated hypoalbuminaemia the free fraction may rise unpredictably.^22,26,59^ This mechanism has not been formally quantified in Fontan cohorts, but the principle is established for other highly protein-bound drugs in the same population.

Vitamin K antagonists inhibit activation of vitamin K-dependent clotting factors II, VII, IX and X alongside protein C and S, with dosing titrated to an INR target of 2–3; however, this range is often unstable in Fontan patients due to FALD-related impairment of hepatic synthesis, which amplifies anticoagulant sensitivity beyond what the INR reliably reflects.^60,61^ A further PK layer compounds this instability: warfarin is highly protein-bound, and hypoalbuminaemia from PLE increases its free fraction and pharmacological effect at any given total plasma concentration.^22,23^ The co-prescription of amiodarone, whose CYP-inhibitory profile and FALD-driven accumulation are detailed in “Liver”, further narrows the therapeutic window by reducing warfarin clearance.^49,50^ These interacting mechanisms are expected to compromise anticoagulant stability. In the available data, this is manifested specifically as prolonged subtherapeutic exposure: more than a quarter of Fontan patients on VKAs in a paediatric cohort spent over 70% of observed time below the therapeutic threshold, independently associated with a 3.5-fold increase in thromboembolic risk.^62,63^ The reciprocal hazard of supratherapeutic excursions and bleeding, although mechanistically plausible given the same sources of variability, was not directly quantified in these cohorts and remains an inference rather than a reported finding. That the network meta-analysis by Van den Eynde *et al*. nonetheless confirmed warfarin's antithrombotic efficacy relative to no prophylaxis (IRR 0.23; 95% CI 0.14–0.37), numerically indistinguishable from aspirin, yet with a substantially inferior bleeding safety ranking (*P*-score 0.292 vs. 0.842), highlights the central tension: population-level efficacy achieved at the cost of a PK profile that makes safe, sustained anticoagulation considerably harder to deliver in this specific population than in any cohort from which these estimates were originally derived.^57^

Direct oral anticoagulants offer a mechanistically appealing alternative, removing the INR monitoring burden from a population committed to lifelong antithrombotic therapy. The PK rationale for caution is, however, built into the Fontan circulation itself. For rivaroxaban, reduced CO and disease-specific anthropometric differences characteristic of Fontan physiology were incorporated into a physiologically based PK model used to support dosing in the UNIVERSE trial.^41^ The model predicted a body weight–based paediatric regimen achieving exposures comparable to 10 mg once daily in adults, which was subsequently confirmed clinically.^41^ Importantly, the model suggested that Fontan physiology did not substantially alter rivaroxaban PKs compared with weight-matched healthy children.^41^ Apixaban data in the Fontan population are now emerging. In the SAXOPHONE trial, weight-adjusted apixaban dosing in 192 children, 67% of whom were post-Fontan, achieved steady-state exposures consistent with adult levels, with composite major and clinically relevant non-major bleeding of 0.8% vs. 4.8% on standard of care, and no thromboembolic events in either arm.^64^ A single-centre real-world series of 62 post-Fontan children confirmed feasibility, with treatment-related thrombosis and bleeding rates of 0.07 and 0.22 per 1000 patient-days respectively, while explicitly identifying co-prescribed CYP3A4 and P-glycoprotein modulators, amiodarone and diltiazem among them, as clinically meaningful determinants of apixaban exposure in this population.^65^ In the network meta-analysis by Van Den Eyden *et al*., NOACs as a class achieved the highest antithrombotic ranking (IRR 0.11; 95% CI 0.03–0.40; P-score 0.921) but also the highest major bleeding point estimate (IRR 1.45; 95% CI 0.28–7.43), non-significant but requiring cautious interpretation given the limited number of patients and the heterogeneity of studies contributing NOAC data.^57^ For dabigatran, no Fontan-specific data exist; its exclusive renal clearance warrants particular caution given the systematic GFR overestimation in this population.^33^

Both major guideline frameworks acknowledge the evidence without resolving it. The 2020 ESC Guidelines confine mandatory anticoagulation to patients with atrial thrombus, arrhythmias, or prior thromboembolism, while explicitly recognizing that universal prophylaxis lacks robust evidence and that practice varies substantially between centres.^8^ The 2025 ACC/AHA Guidelines represent a meaningful shift, upgrading antithrombotic prophylaxis to Class I (LOE B-NR) for all Fontan patients without bleeding contraindications, with either aspirin or anticoagulation considered equally acceptable for primary prevention.^7^ Neither guideline resolves optimal agent selection, nor addresses the PK reality that the Fontan circulation systematically alters the handling of all three drug classes in ways that population-level efficacy estimates alone cannot capture.

### Heart failure therapy

HF is the principal driver of late morbidity and mortality in the Fontan population, yet the pharmacological evidence base remains strikingly sparse. A systematic review of nine eligible studies encompassing conventional and emerging agents found clinical signals to be inconsistent across drug classes and phenotype-dependent in ways that standard dosing frameworks do not anticipate.^66^ The key PK point is that the Fontan circulation does not merely make these drugs less effective but alters their handling, distribution, and elimination, compounding clinical uncertainty.

ACE inhibitors and ARBs carry Class I evidence in HF with reduced ejection fraction (HFrEF), but evidence of benefit in the Fontan population is lacking. A randomized crossover trial of enalapril showed no exercise capacity improvement and reduced cardiac index during exertion in some patients^67^; a 2022 prospective interventional study confirmed this lack of benefit and documented a 21% incidence of clinically significant hypotension.^68^ The PK dimension amplifies these concerns: the Fontan circulation's unique dependence on systemic venous pressure to maintain pulmonary blood flow makes it highly sensitive to reductions in preload, and the PREpArE-Fontan registry documented a direct association between ACE inhibitor use and reduced GFR, underscoring the need for renal surveillance.^69^ Selective use should be restricted to patients with systemic left ventricular systolic dysfunction.

Beta-blockers reduce heart rate, myocardial oxygen demand, and sympathetic tone but carry a specific haemodynamic hazard in Fontan physiology: CO is partially heart-rate dependent given a fixed stroke volume, and chronotropic depression may critically limit output. A randomized crossover trial of carvedilol in patients with predominantly preserved ejection fraction showed no exercise benefit and worsened natriuretic peptide levels; benefit emerged only in a separate reduced ejection fraction cohort, confirming phenotype dependency.^45,66,70^ From a PK standpoint, carvedilol deserves specific attention: its oral bioavailability in healthy subjects is approximately 25% owing to extensive first-pass CYP2D6 and CYP2C9 metabolism, but FALD-driven reduction in hepatic blood flow impairs first-pass extraction, potentially producing two- to three-fold higher peak plasma concentrations than anticipated from standard weight-based dosing, a mechanism that likely explains the excess hypotension rates observed in congenital heart disease series.^24,45^ Routine use in asymptomatic patients is not supported; initiation should be cautious and limited to those with documented ventricular dysfunction or arrhythmia indications.^71,72^

Angiotensin Receptor-Neprylisin Inhibitor (ARNI), holds theoretical appeal in Fontan physiology: neprilysin inhibition augments natriuretic peptide signalling, potentially reducing congestion, while angiotensin receptor blockade attenuates adverse remodelling. FDA approval now extends to patients aged one year and above following PARADIGM-HF.^73^ However, all randomized evidence has been generated in biventricular populations, and the Fontan literature is limited to observational reports.^74^ A single-centre retrospective series of 29 CHD patients, 52% single ventricle, found ARNI associated with significant systolic blood pressure reduction and increases in serum creatinine and potassium; hypotension requiring dose adjustment or discontinuation occurred in 41% overall, rising to 60% in the single-ventricle subgroup.^75^ This is pharmacokinetically interpretable: neprilysin inhibition augments bradykinin and other vasoactive mediators, and the preload-dependent Fontan circuit amplifies any reduction in systemic venous return. Modest functional improvements have been reported in systemic right ventricular failure series but not consistently^76,77^; the ongoing ENTRUST-ACHD HF trial is expected to provide the first structured data.^78^ Until then, ARNI should be reserved for documented systolic dysfunction, initiated at the lowest dose, with close haemodynamic and renal monitoring.

SGLT2 inhibitors were developed as antidiabetic agents but have demonstrated mortality and hospitalization benefits across the HF spectrum in non-congenital populations through a variety of proposed mechanisms.^79^ The Fontan failure phenotype, fluid retention, venous congestion, impaired myocardial energetics particularly in pressure-loaded right ventricles, and a high prevalence of chronic kidney disease (CKD), maps directly onto these proposed mechanisms.^80^ A single-centre series of 14 Fontan patients found significant natriuretic peptide reduction in those with HFrEF, with generally acceptable tolerability, though one patient with pre-existing CKD developed acute kidney injury.^80^ A pharmacokinetically relevant observation from the same cohort was a mean serum creatinine rise of 0.18 mg/dL with a corresponding estimated GFR reduction of 26.3 mL/min/1.73m^2^ after initiation, reflecting tubuloglomerular feedback and requiring vigilance in patients with CO-dependent renal perfusion.^80^ Emerging real-world registry data further support tolerability in this population: echocardiographic improvements in systolic function were observed in the first 100 days of SGLT2i therapy in a single ventricle cohort, with benefit concentrated in patients with reduced systolic function, and a larger multicentre analysis of 33 adult Fontan circulatory failure patients demonstrated a significant 26.5% reduction in NT-proBNP after initiation, in both reduced and preserved ejection fraction phenotypes.^81,82^ The gradual osmotic diuresis characteristic of SGLT2 inhibition, without the acute preload reduction of loop diuretics or the vasodilatory effects of ARNI, may represent a relative pharmacodynamic advantage in this circulation. Prospective randomized trials are underway and are anticipated to provide the first controlled evidence in this space (NCT06762964, NCT05741658).

Across all drug classes, the evidence deficit in Fontan HF reflects not merely insufficient trial volume but a PK problem that existing trials were not designed to detect: standard dosing anchored in biventricular HF cohorts does not account for the simultaneous perturbations imposed by this circulation, and the clinical consequences, drug accumulation, therapeutic failure, and toxicity, are therefore mechanistically predictable.

### Antiarrhythmics

Arrhythmias are a major determinant of adverse outcome in the Fontan circulation, with atrial arrhythmias strongly associated with increased risk of transplantation and death.^83^ Management prioritizes maintenance of sinus or paced atrial rhythm, given the dependence of CO on atrioventricular synchrony.^9^ Antiarrhythmic therapy can serve as first-line treatment, although efficacy is modest and caution is required in the presence of sinus node dysfunction due to bradycardia risk.^9,83^ Class Ic agents are generally avoided because of proarrhythmic potential, while Class III agents are preferred.^83^ However, their use is constrained by PK considerations: sotalol and dofetilide are renally cleared, require dose adjustment based on creatinine clearance, and necessitate monitor initiation due to QT prolongation risk, with renal function frequently overestimated in this population.^83,84^

Amiodarone is the most effective long-term antiarrhythmic agent in this population, particularly when ventricular function is impaired.^51^ Its hepatic metabolism and the resulting drug-interaction profile, detailed in “Liver”, make it the antiarrhythmic whose PK behaviour in the Fontan circulation demands the most explicit scrutiny.^85^ Clinical experience in adults with congenital heart disease documents side-effect rates exceeding 50%.^83^ Thyroid dysfunction predominates, particularly thyrotoxicosis in women post-Fontan and those with low BMI, alongside hepatotoxicity. In one cohort, 30% of patients developed thyrotoxicosis and 14% hypothyroidism.^51^ The hepatotoxic potential is further amplified by pre-existing FALD.^52^ Amiodarone should be approached as a drug requiring an explicit exit strategy, whether catheter ablation, Fontan conversion, or transplantation evaluation, rather than indefinite maintenance. Thyroid, hepatic, and pulmonary monitoring is a pharmacokinetic necessity in this context, not a precautionary formality.^9^

### Pulmonary vasodilators

Pulmonary vasodilation is a physiologically coherent target in the Fontan circulation: passive transpulmonary blood flow is exquisitely sensitive to pulmonary vascular resistance (PVR), and even modest reductions augment ventricular preload and CO without the need for a subpulmonary ventricle.^86^ Phosphodiesterase type 5 inhibitors, endothelin receptor antagonists, and prostacyclin analogues, have been evaluated in randomized trials, with consistently positive but clinically modest results. The 2025 ACC/AHA Guideline acknowledges that these agents may improve exercise performance in select adults with Fontan circulation without elevated ventricular end-diastolic pressure.^7^

The PK behaviour of these agents in Fontan patients demands the same scrutiny applied to antiarrhythmics. Hill et al. conducted a prospective randomized PK trial of oral ambrisentan in 13 children on postoperative day one after Fontan surgery, finding dose-adjusted exposure approximately 3.5-fold higher than in children with idiopathic pulmonary arterial hypertension (PAH) receiving comparable weight-based doses, with typical clearance of 1.00 L/hr/70 kg, substantially below adult PAH reference values. Authors attributed this to FALD-driven CYP enzyme suppression compounded by acute hepatic congestion, the same mechanism responsible for the analogous delayed sildenafil clearance the same group had previously documented in post-Fontan children. Model simulations established that a dose of 0.1 mg/kg approximates the therapeutic exposure of the standard adult 5 mg dose, meaning conventional weight-unadjusted prescribing systematically overexposes this population. Beyond chronic dosing, they demonstrated that even a single ambrisentan dose acutely reduced Fontan pressures (16.8–15.6 mmHg; *P* = 0.01), indexed PVR (2.3 to 1.8 WU·m^2^; *P* = 0.01), and plasma BNP (*P* = 0.046) within three hours, without drug-related adverse events.^87^ For prostacyclin analogues, Chen *et al*.^86^ characterized intravenous treprostinil pharmacokinetics in eight paediatric PAH patients after Fontan surgery, demonstrating a linear dose-concentration relationship and identifying height, not weight or body surface area, as an independent PK covariate alongside dose, accounting for 80.2% of the variance in plasma concentration. Moreover, they found that the stable therapeutic dose in this cohort (mean 65.0 ± 7.1 ng/kg/min) substantially exceeded general PAH reference ranges, with significant reductions in mean pulmonary artery pressure (18.6 ± 3.7 to 15.4 ± 3.5 mmHg; *P* = 0.021), Pp/Ps (0.35 ± 0.11 to 0.22 ± 0.06; *P* = 0.002), and oxygenation index (*P* = 0.028) without serious adverse events.

The available PK evidence demonstrates that hepatic congestion and FALD systematically reduce clearance across this drug class, that Fontan patients require doses that deviate substantially from PAH trial protocols, and that overexposure risk is greatest in the immediate post-operative period and in patients with advanced liver disease. Pulmonary vasodilators should therefore be initiated at the lower end of dosing ranges, titrated against clinical and exercise response, and approached with the same PK vigilance applied to every other drug class prescribed in this circulation.

## Phyiologically based pharmacokinetic modelling

**Figure 2:**
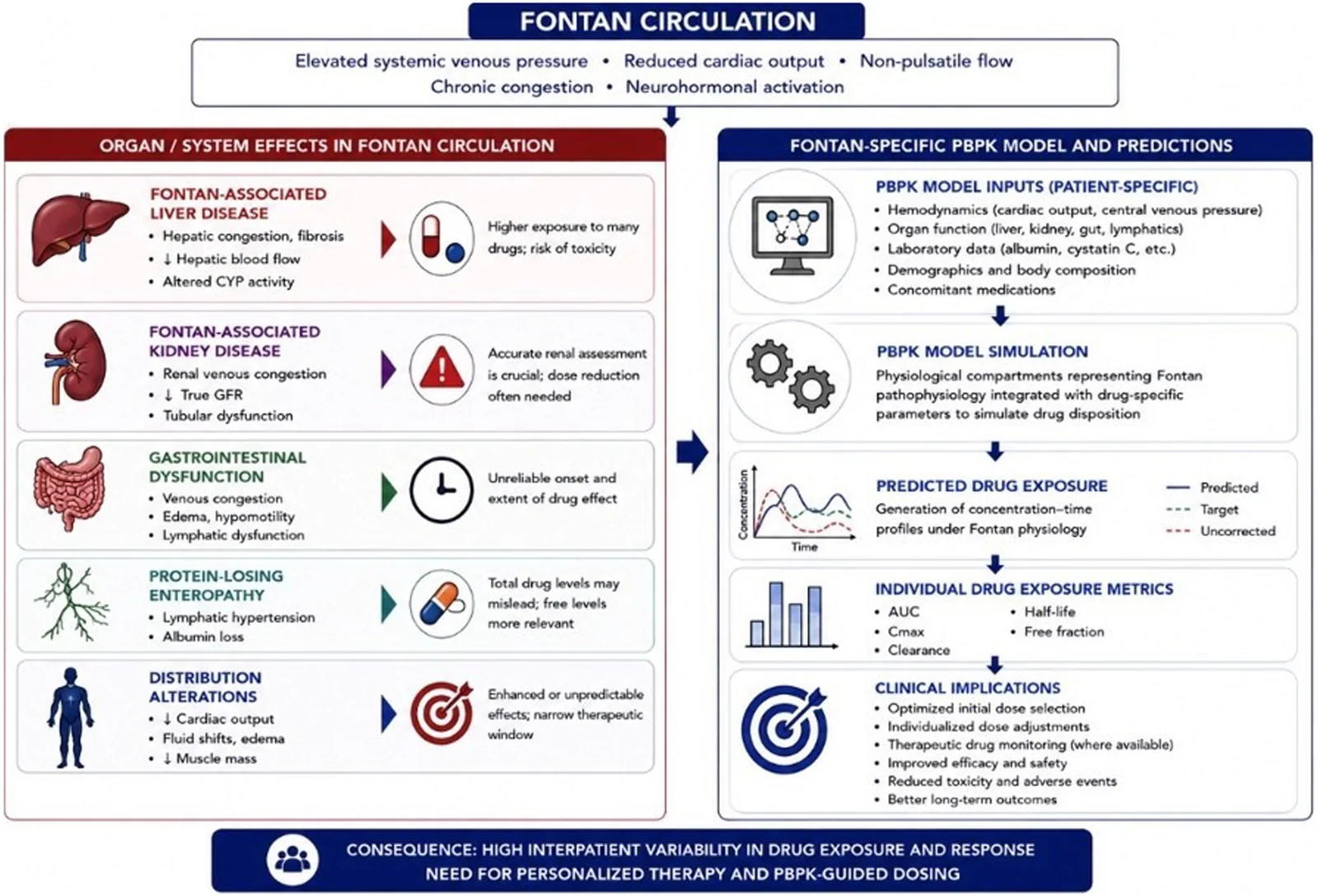
Proposed conceptual framework for a Fontan-specific PBPK model. The left panel summarizes the five principal organ-level perturbations of the Fontan circulation and their predicted pharmacokinetic consequences; the right panel depicts the proposed model architecture, from patient-specific inputs through simulation to individual drug exposure metrics and clinical dosing implications. Concentration-time profiles are schematic and illustrative; no such integrated model has yet been constructed. Created with BioRender.com. *AUC*, area under the concentration-time curve; *Cmax*, maximum plasma concentration; CYP, cytochrome P450; GFR, glomerular filtration rate; PBPK, physiologically based pharmacokinetic.

The PK disturbances in the Fontan circulation operate simultaneously and compound each other in ways that no single reference population can reproduce. PBPK modelling offers the most tractable near-term solution, because it integrates drug-specific physicochemical parameters with quantified organ-level pathophysiology rather than relying on empirical extrapolation from mismatched cohorts. The approach has been validated in Fontan pharmacology by the rivaroxaban UNIVERSE programme, in which a model incorporating reduced cardiac index, paediatric organ volumes, and age-specific CYP enzyme abundance predicted the weight-adjusted dose achieving adult-equivalent anticoagulant exposure, which was subsequently confirmed in the prospective trial.^88^ The component pathophysiology models needed to extend this approach exist in adjacent disease areas: PBPK models for Child-Pugh–stratified hepatic impairment incorporating graded CYP reductions have been reviewed,^89^ and a 2026 comprehensive cirrhosis repository quantifying continuous fibrosis progression and population variability across 30 physiological parameters, validated against known PK parameter ratios in 96% of data points, provides a directly applicable framework for FALD.^90^ No published model has yet combined CO reduction, FALD-calibrated CYP abundance, PLE-adjusted free-drug parameters, and cystatin C–defined GFR into a Fontan virtual population capable of predicting drug exposure across the full spectrum of disease severity encountered in adult practice. This proposed integration of organ-level parameters into a unified Fontan-specific PBPK framework is illustrated in *Figure 2*. Constructing and prospectively validating such a platform, using PK sampling embedded in existing registries such as the Australia and New Zealand Fontan Registry, represents both the most consequential near-term methodological gap and the most direct path from the mechanistic framework of this review to clinically actionable dosing recommendations.

## Clinical implications

In the absence of Fontan-specific PK data for most agents, the following prescribing framework rests on five mechanistic principles applicable across drug classes. These principles derive from PK theory and analogous disease states rather than direct Fontan evidence. Renal function should be estimated by cystatin C rather than creatinine, since the systematic overestimation by creatinine-based equations in Fontan patients, translates directly into underdosing of renal dose-reduction thresholds for sotalol, dabigatran, and digoxin.^27,31^ Hepatic metabolic capacity should be assumed reduced in proportion to FALD severity even when aminotransferases are normal, since histologically advanced disease is consistently present in the absence of transaminase elevation, as established in “the Fontan circulation: organ-system determinants of altered pharmacokinetics” and “Antithrombotic therapy”; hepatically cleared drugs with high extraction ratios and those dependent on CYP3A4 or CYP2C9 should be initiated at the lowest end of the recommended dose range with slow titration. For highly protein-bound drugs in patients with albumin below 30 g/L, total drug concentration measurements are unreliable surrogates for pharmacological effect; dose adjustment guided by clinical response and toxicity monitoring, rather than by target plasma concentrations, is the appropriate strategy in this context. Finally, drug interactions in Fontan patients are additive across all three perturbation mechanisms simultaneously, a patient on amiodarone with FALD and hypoalbuminaemia faces compounding CYP inhibition, reduced intrinsic clearance, and elevated free warfarin fraction, a combination whose net effect on anticoagulant exposure cannot be estimated from any single drug interaction study.

## Future directions

Three priorities define the next phase of Fontan pharmacology research. A Fontan-specific PBPK virtual population, built by integrating published hepatic impairment CYP enzyme reduction frameworks, continuous cirrhosis progression pathophysiology repositories, and paediatric CO adjustment models, would enable prospective dose prediction for agents currently prescribed without PK evidence; the regulatory pathway for model-based labelling when dedicated impairment studies are impractical is established within both FDA and EMA guidance frameworks. Sparse PK sampling embedded into scheduled visits of existing Fontan registries would generate population PK datasets for commonly prescribed agents at minimal additional patient burden, without the ethical and logistical constraints of dedicated PK trials in this small population. Finally, future randomized trials of novel HF therapies must prospectively stratify Fontan patients with embedded PK sampling rather than excluding them by design; the PANORAMA-HF trial restricted enrolment to biventricular physiology, denying the pharmacologically most complex subgroup the exposure-response data that evidence-based dosing requires.^91^

## Conclusion

The Fontan circulation constitutes a distinct PK environment whose complexity current prescribing practice systematically underestimates, and the clinical consequences, drug accumulation, toxicity, and therapeutic failure, are not random but mechanistically predictable. The generation of patients whose survival the Fontan operation made possible now requires the pharmacological rigour that their physiology demands.

## Data Availability

No new data were generated or analysed in support of this research.
